# Anti-Apoptotic Effects of 3,3’,5-Triiodo-L-Thyronine in the Liver of Brain-Dead Rats

**DOI:** 10.1371/journal.pone.0138749

**Published:** 2015-10-05

**Authors:** Rolando A. Rebolledo, Anne C. Van Erp, Petra J. Ottens, Janneke Wiersema-Buist, Henri G. D. Leuvenink, Pamela Romanque

**Affiliations:** 1 Department of Surgery, University Medical Center Groningen, Groningen, The Netherlands; 2 Physiopathology Program, Institute of Biomedical Sciences, Faculty of Medicine, University of Chile, Santiago, Chile; IDIBAPS - Hospital Clinic de Barcelona, SPAIN

## Abstract

**Background:**

Thyroid hormone treatment in brain-dead organ donors has been extensively studied and applied in the clinical setting. However, its clinical applicability remains controversial due to a varying degree of success and a lack of mechanistic understanding about the therapeutic effects of 3,3’,5-Triiodo-L-thyronine (T_3_). T_3_ pre-conditioning leads to anti-apoptotic and pro-mitotic effects in liver tissue following ischemia/reperfusion injury. Therefore, we aimed to study the effects of T_3_ pre-conditioning in the liver of brain-dead rats.

**Methods:**

Brain death (BD) was induced in mechanically ventilated rats by inflation of a Fogarty catheter in the epidural space. T_3_ (0.1 mg/kg) or vehicle was administered intraperitoneally 2 h prior to BD induction. After 4 h of BD, serum and liver tissue were collected. RT-qPCR, routine biochemistry, and immunohistochemistry were performed.

**Results:**

Brain-dead animals treated with T_3_ had lower plasma levels of AST and ALT, reduced Bax gene expression, and less hepatic cleaved Caspase-3 activation compared to brain-dead animals treated with vehicle. Interestingly, no differences in the expression of inflammatory genes (IL-6, MCP-1, IL-1*β*) or the presence of pro-mitotic markers (Cyclin-D and Ki-67) were found in brain-dead animals treated with T_3_ compared to vehicle-treated animals.

**Conclusion:**

T_3_ pre-conditioning leads to beneficial effects in the liver of brain-dead rats as seen by lower cellular injury and reduced apoptosis, and supports the suggested role of T_3_ hormone therapy in the management of brain-dead donors.

## Introduction

Organ transplantation is the treatment of choice for patients with end stage liver disease. However, shortage of organs is one of the most important challenges in the transplantation field [1]. The majority of organs available for transplantation stems from brain-dead donors [2]. Grafts from brain-dead donors, however, have more frequent delayed graft function and higher rejection rates compared to living donors [3–5]. Therefore, research has focused on understanding and treating the pathophysiological processes during brain death (BD) to optimize transplantation outcomes. Until now, the available evidence suggests that the negative effects of BD in organ donation can be explained by hemodynamic instability [6–9], global hormonal failure [10–12], and a systemic inflammatory response led by the innate immune system [13–16].

During the onset of BD, the intracranial pressure (ICP) increases, leading to progressive ischemia of the cerebrum, brain stem, and spinal cord. This increase in ICP triggers a sympathetic response causing systemic hypertension, also called the Cushing reflex [17]. When systemic hypertension fails to maintain cerebral perfusion, ischemia of the brain stem results in failure of the hypothalamus and pituitary. Thereby, cessation of the hypothalamic-pituitary axis negatively affects systemic hormone regulation, seen by changes in levels of vasopressin, cortisol, and adrenocorticotropic and thyroid hormones [18]. Following the onset of BD, plasma levels of free 3,3’,5-Triiodo-L-thyronine (T_3_) fall to 50% of baseline levels within 1 h after BD and become undetectable after 9 h, whereas levels of thyroid stimulating hormone, free thyroxine, reverse T_3_, and cortisol are variable [11, 19].

In the clinical setting, thyroid hormone treatment in brain-dead donors has been extensively studied and applied [11, 19–21]. However, there remains controversy about the effectiveness of thyroid therapy due to varying degrees of success. Moreover, the mechanisms behind the therapeutic effects of thyroid hormones are only beginning to be understood [22]. Even though reports have shown beneficial effects of thyroid hormone replacement on the hemodynamic status of the donor, no conclusive evidence has been published [23–26]. Interestingly, recent data from the Organ Procurement and Transplant Network database show an increase in the use of hormonal replacement therapy including thyroid infusion, which is accompanied by increased organ availability [27, 28]. Moreover, early treatment of potential donors with levothyroxine before the declaration of BD is associated with a significant increase in organs donated per donor and reduced vasoactive support [29, 30]. Thus, improving and expanding the treatment of potential donors can lead to an increase in the number of transplantable organs. These beneficial effects of earlier donor treatment could be explained by protective pre-conditioning effects of thyroid hormones, as seen in models of ischemia/reperfusion (I/R)-injury [31, 32] and regeneration in the liver [33, 34]. In these models, T_3_ pre-treatment reduces hepatic injury and apoptosis, and increases liver cell regeneration. Moreover, T_3_ induces hepatocyte proliferation in rat models *in vitro* and *in vivo*, through reduction of apoptosis and stimulation of liver cell regeneration.

Given the shift towards earlier treatment of potential donors, the current discussion in the literature, and lack of consensus regarding the effectiveness of thyroid therapy, we aimed to look at the effects of thyroid hormone pre-conditioning in the liver using a well-established BD model in rats. We hypothesized T_3_ pre-conditioning could improve liver quality by reducing apoptosis and increasing cellular growth rate. A deeper understanding about the therapeutic effects of T_3_ therapy is required to improve treatment of brain-dead donors and, ultimately, increase organ quality and lower rejection rates.

## Materials and Methods

### Animals and treatment

Male, adult, Fisher F344 rats (250–300 g) were used. All animals received care in compliance with the guidelines of the Institutional Animal Care and Use Committee—Rijksuniversiteit Groningen (IACUC-RUG) according to Experiments on Animals Act (1996) issued by the Ministry of Public Health, Welfare and Sports of the Netherlands. The IACUC-RUG approved this study and all animal care actions. In order to ameliorate suffering, animals were anesthetized using isoflurane during the entire surgical procedure. BD was induced under anesthesia, as previously described [35]. In brief, the procedure was as follows: animals were anesthetized using isoflurane with 100% O_2_. Animals were intubated via a tracheostomy and ventilated as follows: tidal volume of 2.5 ml per stroke, positive end-expiratory pressure of 2 cm of H_2_0, and initial respiratory rate (RR) of 80 cycles per min. RR was corrected based on end tidal CO_2_ levels throughout the experiment. Cannulas were inserted in the femoral artery and vein for continuous mean arterial pressure (MAP) monitoring and volume replacement. Through a frontolateral hole in the skull, a no. 4 Fogarty catheter (Edwards Lifesciences Co, Irvine, USA) was placed in the epidural space and slowly inflated (0.16 ml/min) with saline using a syringe pump (Terufusion, Termo Co, Tokyo, Japan). Inflation of the balloon was terminated once a steep rise in the MAP was noted, reflecting the autonomic storm at the beginning of BD. BD was confirmed by the absence of corneal reflexes and a positive apnea test. Following confirmation of BD, ventilation was continued and anesthesia terminated. MAP was maintained above 80 mmHg. If necessary, colloid infusion with polyhydroxyethyl starch (HAES) 10% (Fresenius Kabi AG, Bad Homburg, Germany) was started (at a maximum rate of 1 ml/h) to maintain a normotensive MAP. Unresponsiveness to HAES administration indicated the start of an intravenous noradrenaline (NA) drip (1 mg/ml). A homeothermic blanket control system was used throughout the experiment. Four hours after the confirmation of BD, blood and urine were collected, after which all abdominal organs were flushed with cold saline. After the flush-out, organs were harvested and tissue samples snap-frozen in liquid nitrogen and stored at -80°C or fixated in 4% paraformaldehyde. Plasma samples and urine were also snap-frozen.

Rats were randomly assigned to each group (n = 7 per group). Sham-operated rats served as controls and were ventilated for 0.5 h under anesthesia. T_3_ (Sigma-Aldrich, cat. nr. T6397) or an equivalent volume of hormone vehicle (0.1 N NaOH, pH = 7.4) was administered intraperitoneally 2 h before the BD period. T_3_ dosage (0.1 mg/kg) was chosen based on previous work [31]. This experiment proved the pre-conditioning effects of T_3_ in a model of hepatic I/R-injury.

The following experimental groups were studied:

- Sham-operated animals pre-treated with vehicle.
- Sham-operated animals pre-treated with T_3_.
- Brain-dead animals pre-treated with vehicle.
- Brain-dead animals pre-treated with T_3_.

### 0.1 Plasma determinations

Plasma levels of alanine transaminase (ALT) and aspartate transaminase (AST) were determined at the clinical chemistry lab of the University Medical Center Groningen according to standard procedures.

### 0.2 RNA isolation and cDNA synthesis

Total RNA was isolated from whole liver sections using TRIzol (Life Technologies, Gaithersburg, USA). Samples were verified for absence of genomic DNA contamination by performing RT-PCR reactions in which the addition of reverse transcriptase was omitted, using Glyceraldehyde 3-phosphate dehydrogenase primers. For cDNA synthesis, 1 *μ*l T11VN Oligo-dT (0,5 *μ*g/*μ*l) and 1 *μ*g mRNA were incubated for 10 min at 70°C and cooled directly after that. cDNA was synthesized by adding a mixture containing 0.5 *μ*l RnaseOUT Ribonuclease inhibitor (Invitrogen, Carlsbad, USA), 0.5 *μ*l RNase water (Promega), 4 *μ*l 5x first strand buffer (Invitrogen), 2 *μ*l DTT (Invitrogen), 1 *μ*l dNTP’s, and 1 *μ*l M-MLV reverse transcriptase (Invitrogen, 200U). The mixture was kept at 37°C for 50 min. Next, reverse transcriptase was inactivated by incubating the mixture for 15 min at 70°C. Samples were stored at -20°C.

### 0.3 Real-Time PCR

Fragments of several genes involved in inflammation (interleukin (IL)-6, IL-1*β*, and monocyte chemotactic protein 1 (MCP-1)), apoptosis (bcl-2-associated X (Bax) and B-cell lymphoma 2 (Bcl-2)), mitosis (Cyclin-D), and oxidative stress (signal transducer and activator of transcription 3 (STAT3) and nuclear transcription factor erythroid 2-related factor 2 (Nrf2)) were amplified with the primer sets outlined in Table 1. Pooled cDNA obtained from brain-dead rats was used as internal reference. Gene expression was normalized with the mean *β*-actin mRNA content. Real-Time PCR was carried out in reaction volumes of 15 *μ*l containing 10 *μ*l of SYBR Green mastermix (Applied biosystems, Foster City, USA), 0.4 *μ*l of each primer (50 *μ*M), 4.2 *μ*l of nuclease free water, and 10 ng of cDNA. All samples were analyzed in triplicate. Thermal cycling was performed on the Taqman Applied Biosystems 7900HT Real-Time PCR System with a hot start for 2 min at 50°C, followed by 10 min at 95°C. The second stage started with 15 s at 95°C (denaturation step) and 60 s at 60°C (annealing step and DNA synthesis). The latter stage was repeated 40 times. Stage 3 was included to detect formation of primer dimers (melting curve) and began with 15 s at 95°C, followed by 60 s at 60°C and 15 s at 95°C. Primers were designed with Primer Express software (Applied Biosystems) and primer efficiencies were tested by a standard curve for the primer pair, resulting from amplification of a serial dilution of cDNA samples (10 ng, 5 ng, 2.5 ng, 1.25 ng, and 0.625 ng) obtained from brain-dead rats. PCR efficiency was 1.8 < *ϵ* < 2.0. Real-time PCR products were checked for product specificity on a 1.5% agarose gel. Results were expressed as 2^−△△*CT*^ (CT: Threshold Cycle).

**Table 1:** Primer sequences used for Real-Time PCR.

Gene	Primers	Amplicon size (bp)
IL-1*β*	5’- CAGCAATGGTCGGGACATAGTT-3’ 5’-GCATTAGGAATAGTGCAGCCATCT-3’	75
IL-6	5’-CCAACTTCCAATGCTCTCCTAATG-3’ 5’-TTCAAGTGCTTTCAAGAGTTGGAT-3’	89
MCP-1	5’-CTTTGAATGTGAACTTGACCCATAA-3’ 5’-ACAGAAGTGCTTGAGGTGGTTGT-3’	78
Bax	5’-GCGTGGTTGCCCTCTTCTAC-3’ 5’-TGATCAGCTCGGGCACTTTAGT-3’	74
Bcl-2	5’-CTGGGATGCCTTTGTGGAA-3’ 5’-TCAGAGACAGCCAGGAGAAATCA-3’	70
Cyclin D	5’-CAGCATTGTGCTAATGTAAAGCCAG-3’ 5’-TGGGACGCCTCAGCTAAGCT-3’	103
STAT3	5’-TTCGAGGCAGTCATTGCTCTC-3’ 5’-ACGCCTTGCCTTCCTAAATACC-3’	97
Nrf2	5’-CAACGAAGCTCAGCTTGCATTA-3’ 5’-TCCTACAGTTCTGAGCGGCAAC-3’	76

### 0.4 Immunohistochemistry and histopathological evaluation

Immunohistochemistry was performed on 3 *μ*m sections of paraffin-embedded liver samples to detect cleaved caspase 3 (cC3), Ki-67, and heme oxygenase 1 (HO-1) positive cells. The active form (cleaved) of caspase 3 was assessed to study apoptosis. Ki-67 is a cellular marker used to study proliferation [36], whereas HO-1 is an oxidative stress response marker [37]. Sections were deparaffinized in a sequence of xylene, alcohol, and water, and subjected to heat-induced antigen retrieval by microwave heating in 1 mM EDTA buffer (pH = 8.0, for cC3), 10 mM citrate buffer (pH = 6.0, for Ki-67), or overnight incubation in 0.1 M Tris/HCl buffer at 80°C (pH = 9.0, for HO-1). Next, sections were stained with cC3 pAb (Cell Signaling, cat. nr. 9661, 100 x diluted in 1% BSA/PBS), Ki-67 pAb (Abcam, cat. nr. 15580, 500 x diluted in 1% BSA/PBS + 1% Triton) or HO-1 mAb (Stressgen, cat. nr. ADI-OSA-111F, 250 x diluted in 1% BSA/PBS) using an indirect immunoperoxidase technique. Endogenous peroxidase was blocked using H_2_O_2_ 0.3% in phosphate-buffered saline for 30 min. After thorough washing, sections were incubated with horseradish peroxidase-conjugated goat anti-rabbit IgG as a secondary antibody for 30 min (Dako, Glostrup, Denmark, cat. nr. P0448), followed by rabbit anti-goat IgG as a tertiary antibody for 30 min (Dako, Glostrup, Denmark, cat. nr. P0449). The reaction was developed using DAB as chromogen and H_2_O_2_ as substrate. Sections were counterstained using Mayer hematoxylin solution (Merck, Darmstadt, Germany). Negative antibody controls were performed. Localization of immunohistochemical staining was assessed by light microscopy. For each tissue section, positive cells per field were counted by two blinded researchers in 10 microscopic fields of the tissue at 20 x magnification. Results were presented as number of positive cells per field. Additional HO-1 intensity quantification was performed using Aperio Image Scope (Leica Biosystems, Nussloch, Germany), with algorithm positive pixel count v9 to perform the analysis. Data are presented as total intensity per area (*mm* ^2^).

The extent of hepatic injury was determined in 3 *μ*m sections of hematoxylin-eosin (HE) stained, paraffin-embedded slides, which were blindly scored by a pathologist. Slides were scored for conservation of cytoarchitecture and lobule organization, hepatocyte appearance, localization of portal and central veins, and amount of necrosis.

### Statistical analysis

Due to the two-factorial design of the experiment, the two-way ANOVA test was used to analyze the results (SPSS Statistics 20, IBM Software, NY, USA). Data were checked for a normal distribution and equality of variances of the dependent variable across groups using Levene’s test of equality of error variances (indicated by p > 0.05). Data transformations were applied if these assumptions were not met, after which data were normally distributed and variances of the dependent variable equal across groups. This allowed us to use the two-way ANOVA test despite small group sizes. For AST and the Bax/Bcl-2 ratio, data did not meet these assumptions. For these parameters, the Kruskal-Wallis test was performed to analyze between the four experimental groups, followed by the Mann-Whitney test to compare between two groups individually (Prism 5.0, GraphPad Software, CA, USA). All statistical tests were 2-tailed and p < 0.05 was regarded as significant. Results are presented as mean ± SD (standard deviation).

## Results

Induction of BD showed a uniform pattern in alterations of the MAP consistent with earlier reports, with a mean time of BD declaration of 33.26 ± 3.45 min. The MAP of all 28 animals was kept above 80 mmHg during the experiment (S1 Fig). Comparing the BD groups, vehicle-treated animals received 0.91 ± 0.19 ml of HAES 10% to sustain a MAP above 80 mmHg versus 0.78 ± 0.27 ml in the T_3_-treated group, not reaching statistical difference (p = 0.50). The amount of NA used in the BD groups was 1.03 ± 1.25 ml in the vehicle-treated and 0.23 ± 0.34 ml in the T_3_-treated group (p = 0.16, S2 Fig).

**Fig 1:**
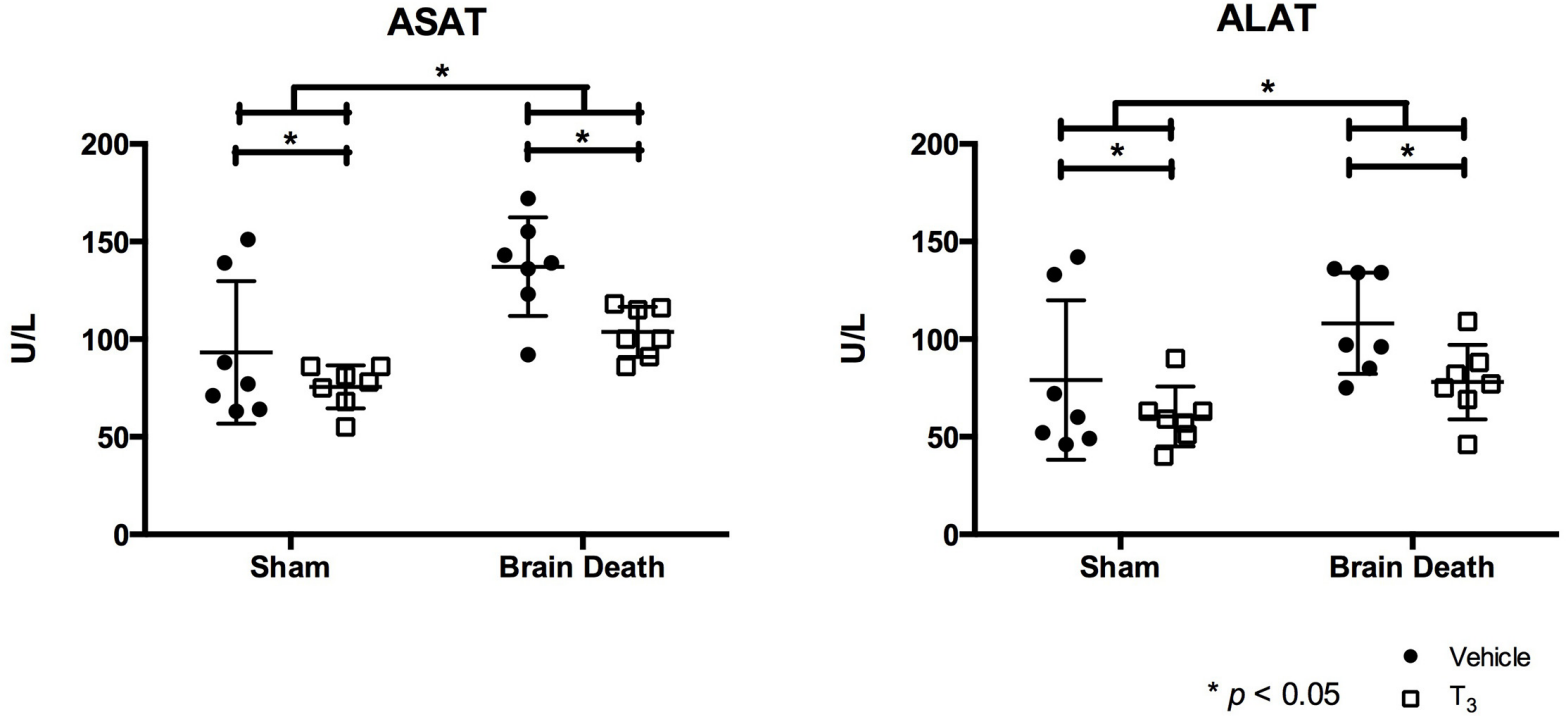
T_3_ pre-treatment attenuated brain death-induced hepatic injury. Plasma levels of AST and ALT were analyzed following 4 h of BD in the BD groups and 0.5 h of ventilation in the sham groups. Groups were pre-treated with either vehicle or T_3_. Results are presented as mean ± SD, n = 7 per group (* p < 0.05).

Plasma levels of liver enzymes AST and ALT were measured as liver cell injury markers. AST and ALT levels were significantly higher in brain-dead animals compared to sham animals (p < 0.05). Yet, T_3_-treatment in brain-dead as well as sham animals significantly reduced AST and ALT levels compared to vehicle-treated animals (p < 0.05, Fig 1). A histological analysis of HE stained, paraffin-embedded slides revealed no recognizable injury pattern, conserved cytoarchitecture and lobule organization, normal hepatocyte appearance and localization of portal and central veins, and no necrosis in the liver of brain-dead animals, all of which were unaffected by T_3_-treatment.

**Fig 2:**
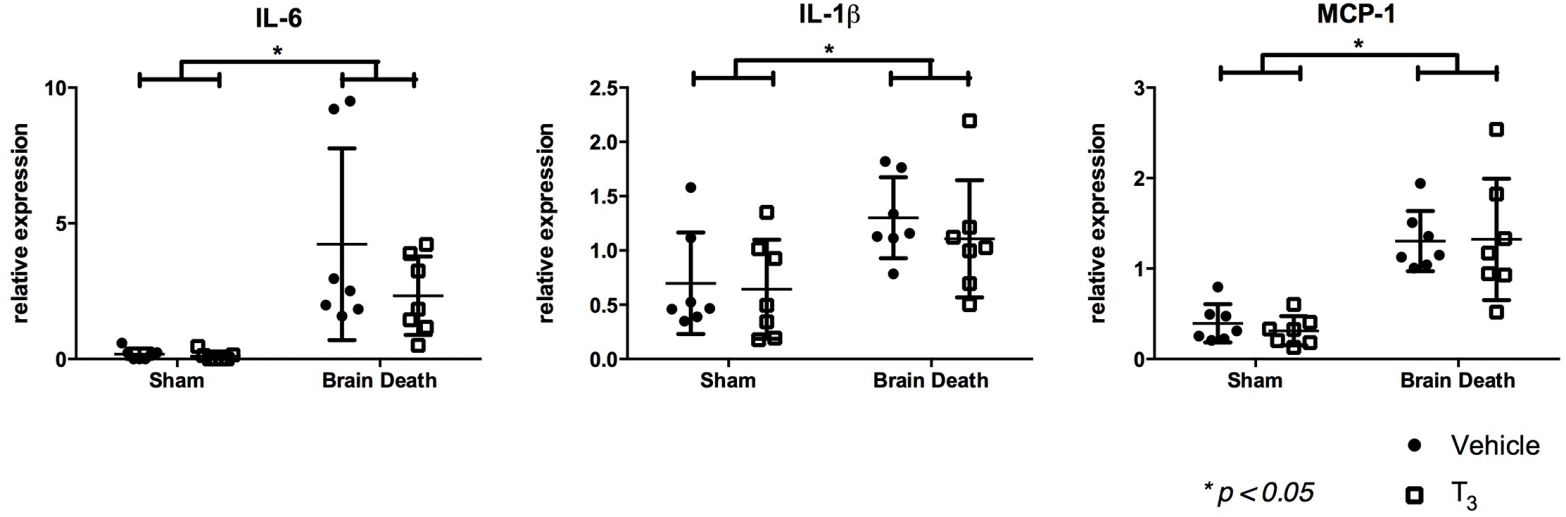
T_3_ pre-treatment did not mitigate hepatic inflammation following BD. Hepatic, relative mRNA expression of inflammation-related genes IL-6, IL-1*β*, and MCP-1 was analyzed by real-time PCR, following 4 h of BD in the BD groups and 0.5 h of ventilation in the sham groups. Groups were pre-treated with either vehicle or T_3_. Data are presented as mean ± SD, n = 7 per group (* p < 0.05).

In order to assess the hepatic inflammatory response in brain-dead animals, inflammatory genes interleukin IL-6, MCP-1, and IL-1*β* were analyzed. The relative expression of IL-6, MCP-1, and IL-1*β* were all significantly increased in brain-dead animals compared to sham animals (p < 0.001, p < 0.001, and p < 0.05 respectively). No differences were found between T_3_-treated and vehicle-treated animals (Fig 2).

**Fig 3:**
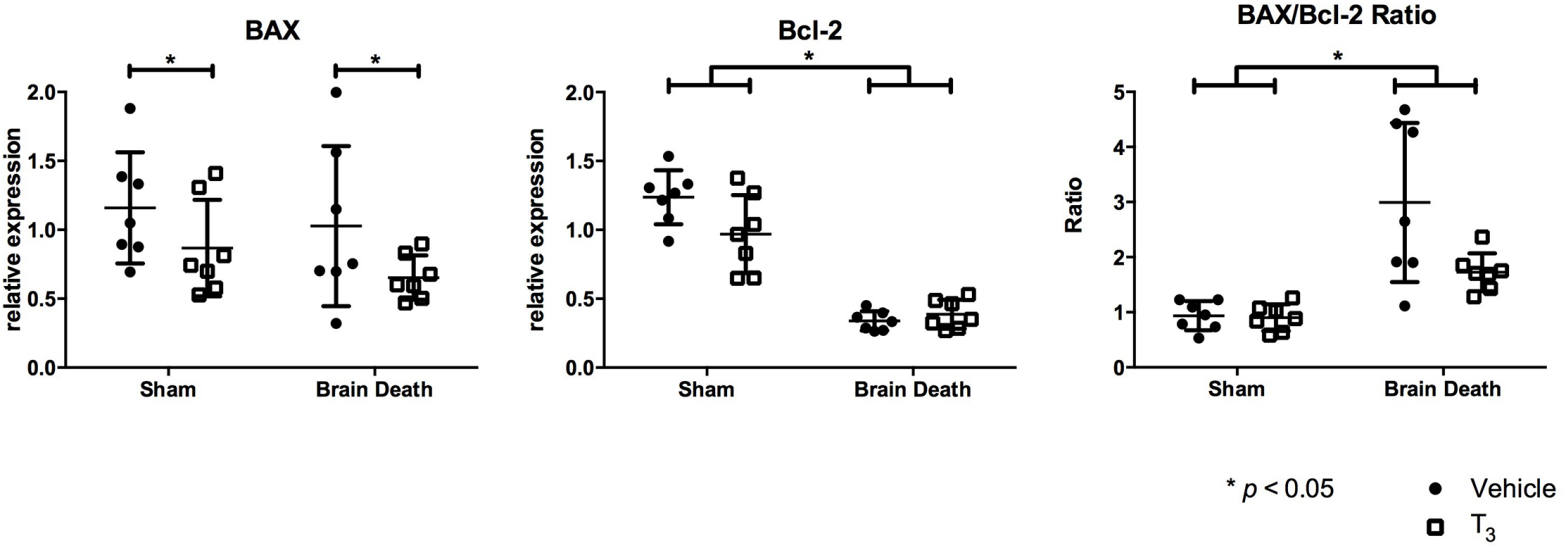
T_3_ pre-treatment attenuated hepatic apoptosis. Hepatic, relative mRNA expression of pro-apoptotic gene Bax and anti-apoptotic gene Bcl-2, and the Bax/Bcl2 mRNA ratio were analyzed by real-time PCR, following 4 h of BD in the BD groups and 0.5 h of ventilation in the sham groups. Groups were pre-treated with either vehicle or T_3_. Data are presented as mean ± SD, n = 7 per group (* p < 0.05).

**Fig 4:**
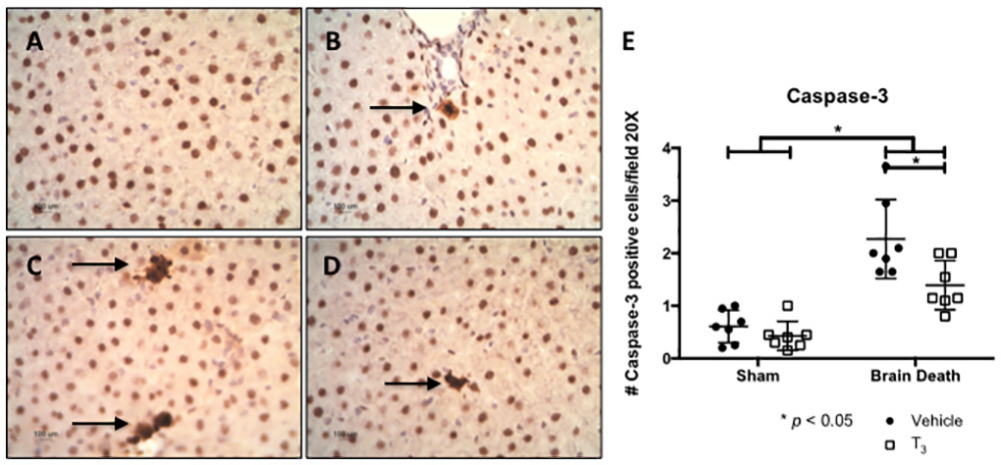
T_3_ pre-treatment attenuated brain death-induced apoptosis in the liver. Analyses performed following 4 h of BD in the BD groups and 0.5 h of ventilation in the sham groups. (A), (B), (C), and (D) Hepatic, cleaved Caspase-3 (cC3) immunohistochemistry staining at 20 x original magnification from (A) sham + vehicle, (B) sham + T_3_, (C) BD + vehicle and (D) BD + T_3_-treated rats. The sections are representative of 7 independent rats per group. Black arrows show examples of positive cells. (E) cC3 protein quantification in the liver, shown as the number of positive cells in the liver per field at 20 x original magnification, counted twice in a blinded fashion.

To evaluate possible anti-apoptotic effects, the relative expression of complementary genes Bcl-2 and Bax, the Bax/Bcl2 ratio, and the presence of cC3 in the liver tissue were analyzed (Fig 3). The relative expression of apoptosis promotor Bax was not significantly different in brain-dead animals compared to sham animals. However, T_3_ treatment significantly lowered Bax gene expression compared to vehicle treatment in both sham and brain-dead rats (p < 0.05). Relative expression of apoptosis inhibitor Bcl-2 was significantly decreased following BD (p < 0.001) but did not change in T_3_-treated animals. The Bax/Bcl-2 ratio shows the balance between pro-apoptotic and anti-apoptotic genes. This ratio was significantly increased in brain-dead animals, indicating a pro-apoptotic tendency (p < 0.01). However, the Bax/Bcl-2 ratio showed no change in T_3_-treated, brain-dead animals compared to vehicle-treated animals (p = 0.053). cC3 is a protein that acts downstream of Bax and Bcl-2 control and is a key player in the execution phase of cellular apoptosis. The number of cC3 positive cells in the liver of brain-dead animals was higher compared to sham animals (p < 0.05), and was significantly reduced following T_3_ treatment in both sham and brain-dead animals (p < 0.01, Fig 4).

**Fig 5:**
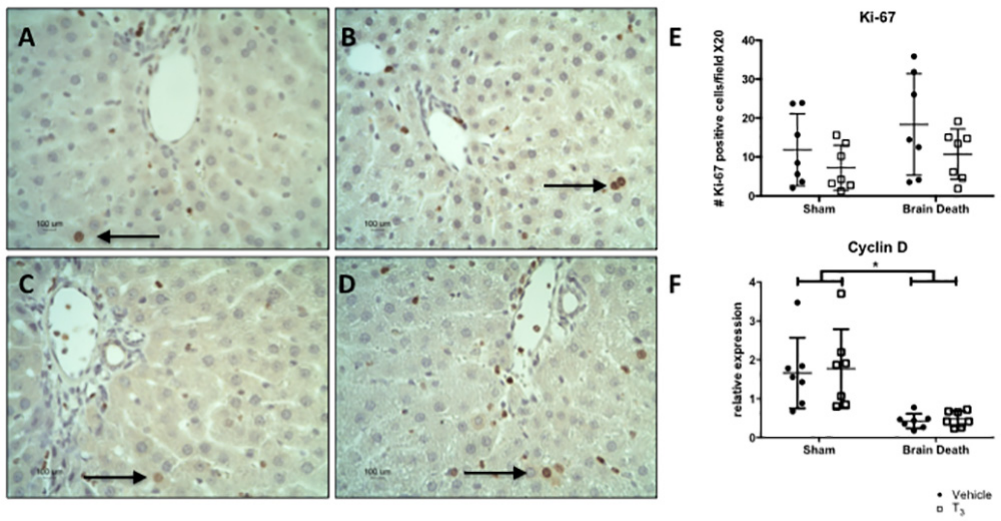
T_3_ pre-treatment did not give rise to mitotic effects in the liver. Analyses performed following 4 h of BD in the BD groups and 0.5 h of ventilation in the sham groups. (A), (B), (C), and (D) Immunohistochemistry staining of cellular proliferation marker Ki-67 at 100 x original magnification in the liver from (A) sham + vehicle, (B) sham + T_3_, (C) BD + vehicle, and (D) BD + T_3_-treated rats. The sections are representative of 7 independent rats per group. (E) Ki-67 protein quantification in the liver, shown as the number of positive cells in the liver per field at 20 x original magnification, counted twice in a blinded fashion. Black arrows show examples of positive cells. (F) Hepatic, relative mRNA expression of pro-mitotic gene Cyclin-D was analyzed by real-time PCR. Data are presented as mean ± SD, n = 7 per group (* p < 0.05).

To determine whether T_3_ treatment also resulted in pro-mitotic effects, the relative expression of Cyclin-D and presence of Ki-67 positive cells were analyzed. Cyclin D is involved in cell cycle progression and Ki-67 is a marker of proliferation as it is present in all proliferating cells [38]. The number of Ki-67 positive liver cells did not change following BD or T_3_ treatment (Fig 5A–5E). Relative expression of Cyclin-D in liver tissue was significantly lower in brain-dead compared to sham animals (p < 0.001), but did not change following T_3_ treatment (Fig 5F).

**Fig 6:**
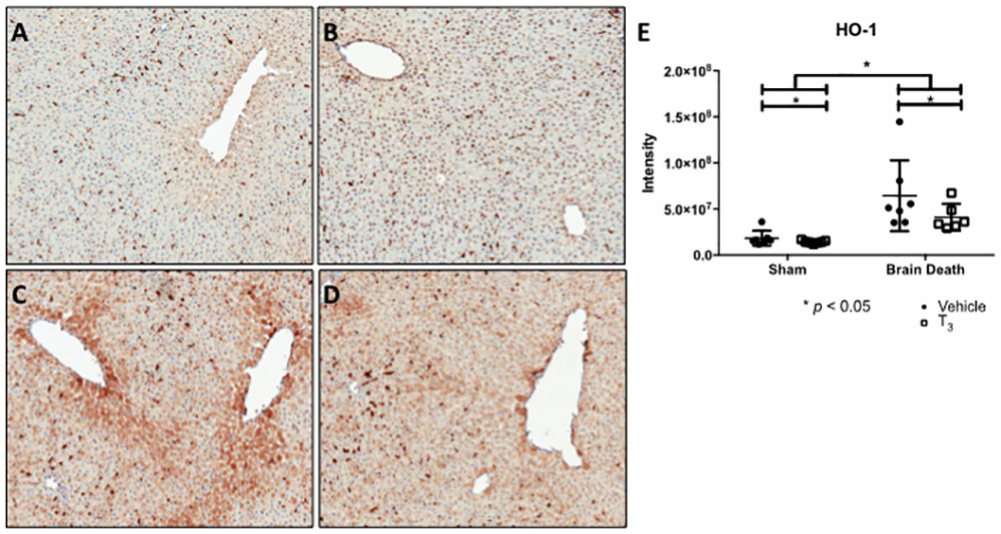
T_3_ pre-treatment reduced brain death-induced oxidative stress in the liver. Analyses performed following 4 h of BD in the BD groups and 0.5 h of ventilation in the sham groups. (A), (B), (C), and (D) Immunohistochemistry staining of cellular oxidative stress marker HO-1 at 100 x original magnification in the liver from (A) sham + vehicle, (B) sham + T_3_, (C) BD + vehicle and (D) BD + T_3_-treated rats. The sections are representative of 7 independent rats per group. (E) HO-1 protein quantification in the liver, counted using Aperio Image Scope. Data are presented as total intensity per area (*mm* ^2^), n = 7 per group (* p < 0.05).

**Fig 7:**
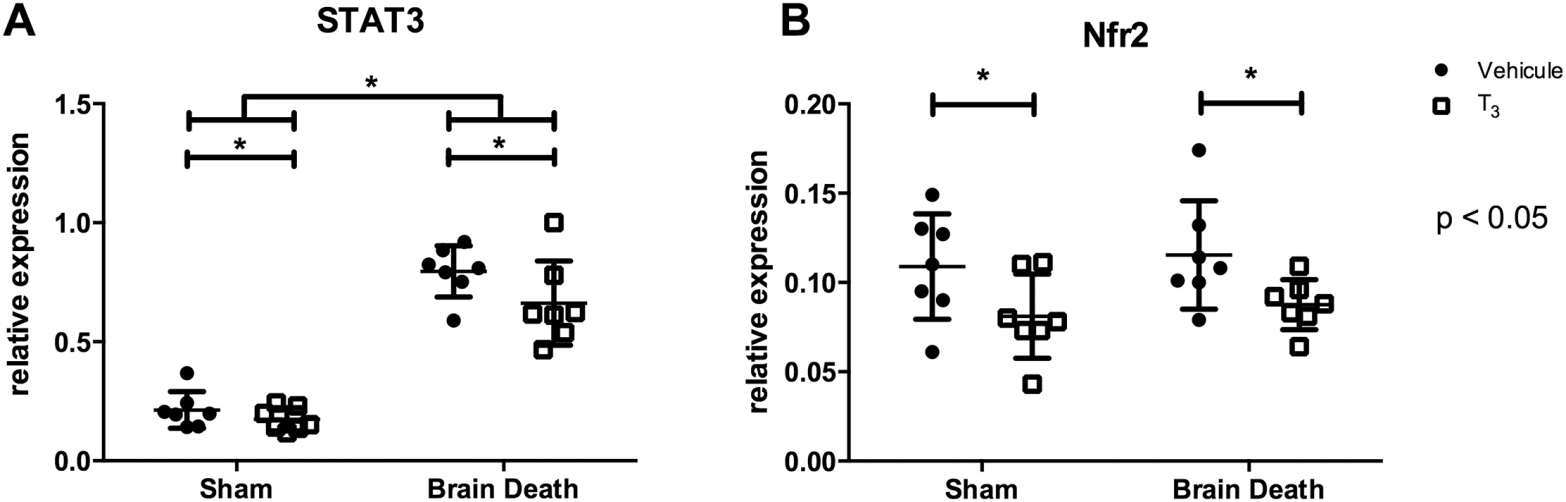
T_3_ pre-treatment reduced brain death-induced oxidative stress in the liver. Hepatic, relative mRNA expression of oxidative stress-related markers STAT3 and Nrf2 was analyzed by real-time PCR, following 4 h of BD in the BD groups and 0.5 h of ventilation in the sham groups. Groups were pre-treated with either vehicle or T_3_. Data are presented as mean ± SD, n = 7 per group (* p < 0.05).

To evaluate the oxidative stress response in the liver, STAT3 and Nrf2 gene expression and HO-1 protein levels were studied. Levels of oxidative stress marker HO-1 were significantly increased in the BD groups (p < 0.0001), however, this was significantly decreased in T_3_-treated animals (p < 0.05, Fig 6A–6E). The relative expression of STAT3 was significantly increased in the BD groups (p < 0.001), yet T_3_ treatment significantly lowered its relative expression (p < 0.05, Fig 7A). The relative expression of Nrf2 was decreased in both (sham and BD) groups treated with T_3_, but no change was observed in brain-dead compared to sham animals (Fig 7B).

## Discussion

To solve the growing disparity between organ supply and demand, hormonal therapy is widely used as a way to improve organ quality and expand the donor pool. Regarding the treatment of brain-dead donors, there is an ongoing discussion about the effectiveness of thyroid hormone therapy together with a shift to start treatment earlier, even before the declaration of BD. In light of this, this present study demonstrates that T_3_ administration before the induction of BD decreases liver cell injury and apoptosis in brain-dead rats, suggesting liver function improves following T_3_ treatment. We show that T_3_ therapy reduces apoptosis initially on a transcriptional level, as well as during the execution phase of apoptosis in brain-dead rats, by reduction of cC3. This is the first time that these anti-apoptotic effects of T_3_ pre-conditioning have been shown in the BD setting. Interestingly, the mechanism through which T_3_ exerts these anti-apoptotic effects remains intact in brain-dead donors and is achieved in a short time frame. Pantos et al. also showed that administration of T_3_ limited apoptosis in a Langendorff-perfused heart model in rats, when given at the time of reperfusion [39]. But, others have shown similar pre-conditioning effects in much larger time frames [31, 32, 40]. In the BD setting, thyroid hormone therapy could thus represent a valid therapeutic opportunity to improve organ quality before donation.

Besides reducing apoptosis, T_3_ also has the capacity to regenerate hepatocytes directly by inducing mitosis [32]. In our model, T_3_ therapy did not alter regulation of cell cycle progression or expression of cellular growth markers [38]. In healthy liver cells, T_3_ has been shown to activate cyclin D within 2–4 h after administration [41]. Yet, in a model of partial hepatectomy, T_3_ pre-treatment increased cyclin D levels and Ki-67 positive cells no earlier than 12 h post-surgery [32]. This suggests pro-mitotic effects are not observed in our study either due to the short therapeutic time frame or due to a differential response to T_3_ in the liver following BD. In the clinical setting, the time frame from the declaration of BD until the actual transplantation more closely resembles models that support the idea that T_3_ induces pro-mitotic effects [33, 34], Further clinical research into the pro-mitotic effects of T_3_ needs to be performed to elucidate this topic.

As mentioned, the available evidence shows that BD causes detrimental pathophysiological changes in the donor. BD is characterized as a systemic inflammatory state, seen by a rise in circulating cytokines levels, including IL-6, IL-10, and MCP-1. This inflammatory environment permits an influx of inflammatory cells in the kidneys, liver and lungs, which triggers a local inflammatory and apoptotic response. Even though apoptosis was reduced in the T_3_-treated animals in our model, this was not accompanied by alterations in inflammation. Thyroid hormone treatment has also been shown to improve BD-induced hemodynamic instability and reduce the amount of vasoactive agents required to treat brain-dead donors [23]. However, beneficial effects of T_3_ therapy in this model were not accompanied by improved hemodynamics or reduced vasoactive support.

These results suggest that the beneficial effects of T_3_ pre-conditioning may not be explained by improvements in hemodynamic or inflammatory status in the brain-dead donor. Interestingly, most of the clinical trials concluding that T_3_ therapy as part of the BD donor management does not confer beneficial effects, have focused on hemodynamic parameters as the primary outcome measurement [23, 26, 42]. However, we now show that T_3_ benefits the liver not hemodynamically, but functionally by reducing liver cell injury and apoptosis. Furthermore, T_3_ has been shown to induce a heat-production response, associated with increased oxygen consumption and reactive oxygen species (ROS) generation in the liver [43, 44]. Through this mechanism, T_3_ induces a transient rise in ROS production in the Küpffer cells, resulting in the upregulation of redox-sensitive transcription factors such as STAT3 [45, 46], as well as activation of hepatic Nrf2 [47, 48]. Through induction of transient oxidative stress by means of these pathways, T_3_ prepares the liver for future injury. In our model, T_3_-treatment significantly lowered STAT3 and Nrf2 expression, as well as HO-1 protein levels after 4 h of BD. This suggests T_3_ treatment successfully pre-conditioned the liver against oxidative stress induced by BD pathophysiology. Further research into this mechanism during different time points throughout the BD period is needed to confirm this assumption. Finally, we understand that expansion of the time frame of the experiment is required to shed more light on long-term pre-conditioning effects of T_3_-therapy, for example by combining this model with an isolated perfused liver or transplantation model.

In conclusion, data presented in this study show protective pre-conditioning effects of T_3_ therapy in the liver of brain-dead rats. These promising results were achieved in a relatively short time frame, supporting the notion that T_3_ hormone therapy could be a valid therapeutic opportunity to improve organ quality before donation. Moreover, as organ storage methods are rapidly shifting from static cold storage to *ex vivo* perfusion methods, T_3_ therapy could potentially be given during the organ preservation period, thereby expanding the therapeutic time frame. Undoubtedly, more research needs to be performed to understand possible mechanisms involved as well as clinical applicability. However, these results suggest a promising role for T_3_ hormone therapy as part of BD donor management, with the ultimate goal to improve transplantation outcomes.

## Supporting Information

S1 Fig T_3_ treatment did not affect the blood pressure profile of rats during 4 h of brain death. The graph represents the mean arterial pressure in mmHg, measured by intravenous cannulation of the left femoral artery. The record started with the BD induction, considering time “0” as the end of BD induction and the start of the BD period. The blood pressure profile did not significantly differ between the vehicle-treated (represented by the continuous black line) and the T_3_-treated group (represented by the green continuous green line) (n = 7 per group).
(TIF)

S2 Fig T_3_ treatment did not lower amounts of vasoactive agents used during 4 h of brain death in rats. Amounts of polyhydroxyl starch (HAES) and noradrenaline (NA, 1 mg/ml) given to brain-dead rats during the 4 h experimental procedure to maintain a MAP above 80 mmHg did not significantly differ between T_3_- and sham-treated animals (p = 0.50 and p = 0.16, respectively). Results are presented as mean ± SD (n = 7 per group).
(TIF)
