# Supplementary figures and images for: Anti-Apoptotic Effects of 3,3’,5-Triiodo-L-Thyronine in the Liver of Brain-Dead Rats
Source: PLoS One. 2015 Oct 5;10(10):e0138749. doi: 10.1371/journal.pone.0138749 (PMC4593580; doi:10.1371/journal.pone.0138749)

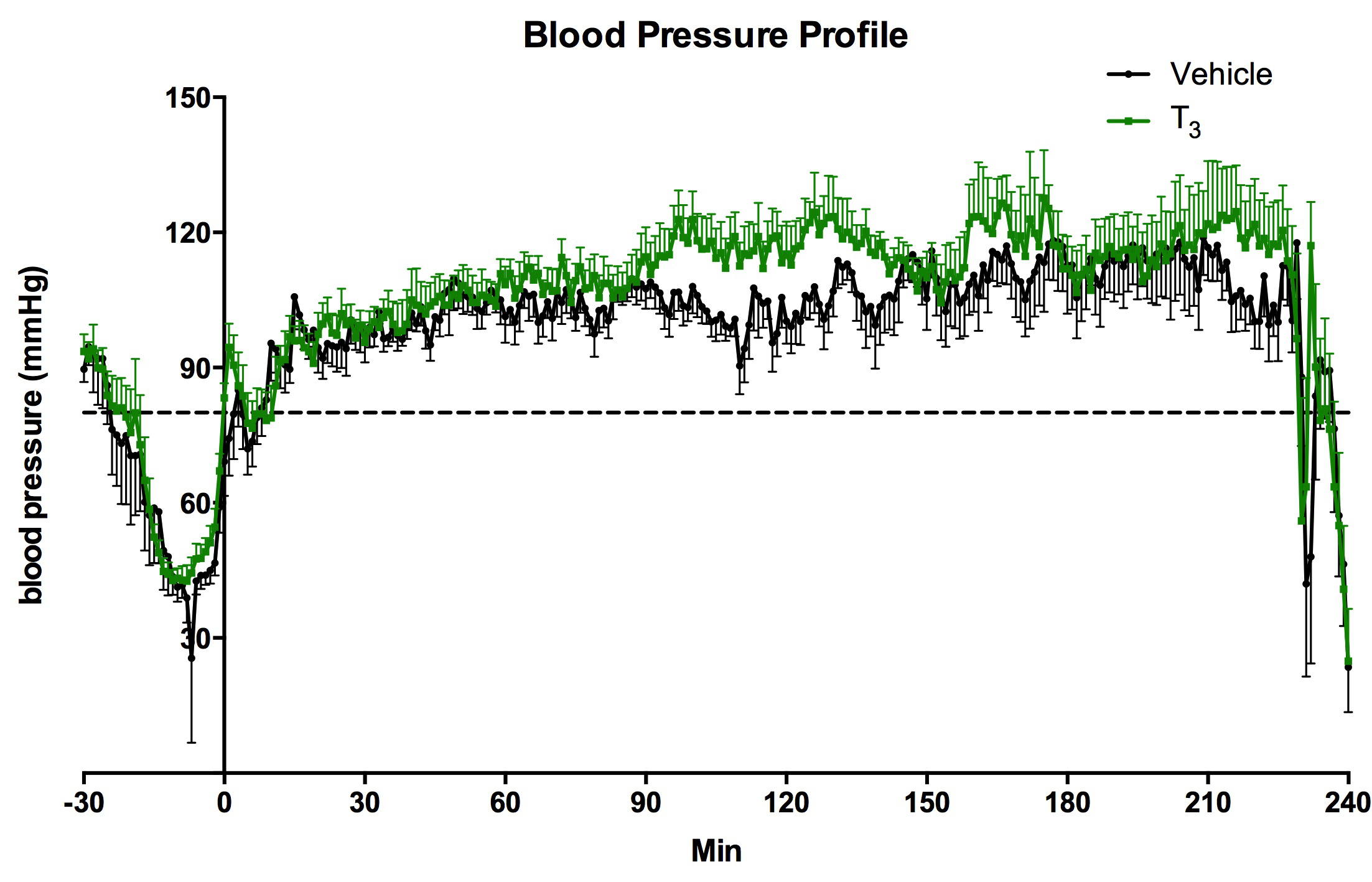

Supplement: S1 Fig — The graph represents the mean arterial pressure in mmHg, measured by intravenous cannulation of the left femoral artery. The record started with the BD induction, considering time “0” as the end of BD induction and the start of the BD period. The blood pressure profile did not significantly differ between the vehicle-treated (represented by the continuous black line) and the T3-treated group (represented by the green continuous green line) (n = 7 per group). (TIF) [file pone.0138749.s001.tif]

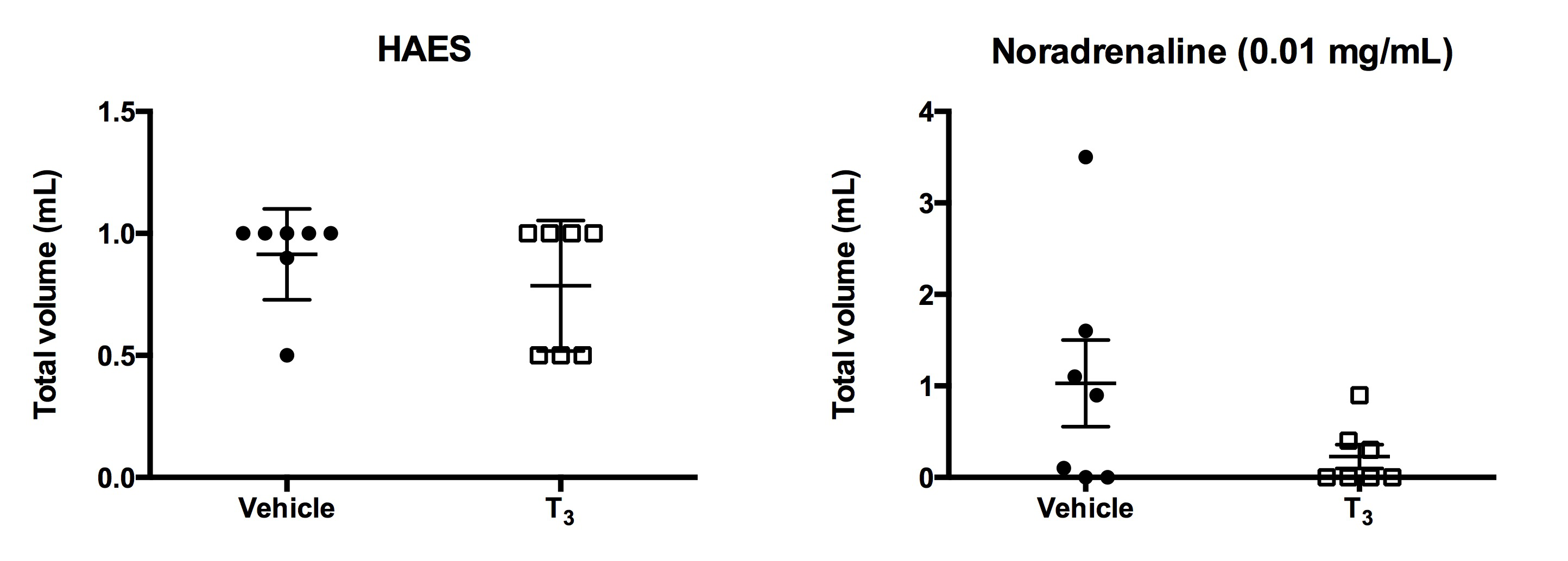

Supplement: S2 Fig — Amounts of polyhydroxyl starch (HAES) and noradrenaline (NA, 1 mg/ml) given to brain-dead rats during the 4 h experimental procedure to maintain a MAP above 80 mmHg did not significantly differ between T3- and sham-treated animals (p = 0.50 and p = 0.16, respectively). Results are presented as mean ± SD (n = 7 per group). (TIF) [file pone.0138749.s002.tif]
